# Data challenges for future plant gene editing: expert opinion

**DOI:** 10.1007/s11248-021-00264-9

**Published:** 2021-06-09

**Authors:** Rim Lassoued, Diego M. Macall, Stuart J. Smyth, Peter W. B. Phillips, Hayley Hesseln

**Affiliations:** 1 Department of Agricultural and Resource Economics, University of Saskatchewan, 51 Campus Drive, Saskatoon, SK S7N 5A8 Canada; 2 The Johnson Shoyama Graduate School of Public Policy, University of Saskatchewan, 101 Diefenbaker Place, Saskatoon, SK S7N 5B8 Canada

**Keywords:** Big data, Data management, Food security, Harmonization, Innovation, Privacy

## Abstract

Agricultural data in its multiple forms are ubiquitous. With progress in crop and input monitoring systems and price reductions over the past decade, data are now being captured at an unprecedented rate. Once compiled, organized and analyzed, these data are capable of providing valuable insights into much of the agri-food supply chain. While much of the focus is on precision farming, agricultural data applications coupled with gene editing tools hold the potential to enhance crop performance and global food security. Yet, digitization of agriculture is a double-edged sword as it comes with inherent security and privacy quandaries. Infrastructure, policies, and practices to better harness the value of data are still lacking. This article reports expert opinions about the potential challenges regarding the use of data relevant to the development and approval of new crop traits as well as mechanisms employed to manage and protect data. While data could be of great value, issues of intellectual property and accessibility surround many of its forms. The key finding of this research is that surveyed experts optimistically report that by 2030, the synergy of computing power and genome editing could have profound effects on the global agri-food system, but that the European Union may not participate fully in this transformation.

## Introduction

Agriculture has steadily adopted a plethora of technological innovations, which are needed now more than ever if global food security issues are to be adequately addressed. Correlated with the revolution in data generation, novel plant breeding is paving the way for new opportunities in crop genetic improvement on a wide-scale and at a lower cost. A new breeding technology increasingly being used, gene editing—spearheaded by clustered regularly interspaced short palindromic repeats (CRISPR/Cas)—is optimistically expected by the research community to enhance global agricultural productivity (Zhu et al. 2020; Lassoued et al. 2018a; Kim and Kim 2019). Experts have already expressed opinions about its benefits (Lassoued et al. 2019a; Qaim 2020; Eshed and Lippman 2019), its risks (Lassoued et al. 2019b; Zhao and Wolt 2017), and how it should be regulated (Lassoued et al. 2020; Whelan and Lema 2019; Eriksson et al. 2019; Wolt et al. 2016; Wolt and Wolf 2018; Fritsch 2019; Gatica-Arias 2020; Smyth 2019a, b; Cavaliere et al. 2019). High-throughput technologies are generating massive heterogeneous data at each stage of the breeding pipeline. Integration, manipulation and interpretation of large volumes of data are increasingly becoming automated and digitized in research and on the farm. Generation of scientific knowledge is profoundly and rapidly changing. Hypothesis-driven research is shifting to data-driven research where new plant genomes can be sequenced and annotated in a matter of days and some farmers are able to generate gigabytes of new data with each field they cultivate (Stevens 2013). The main challenge facing modern plant breeding programs is how to integrate these large volumes of genomic, phenotypic and environmental data to inform variety development decisions (Kuriakose et al. 2020; Zhao et al. 2019). The increased complexity and volume of breeding data make sophisticated analytical tools, new storage systems, and data management facilities for information integration a vital requirement in the agriculture industry (Heckenberger et al. 2008; Kuriakose et al. 2020).

As these tools become more useable, the research community will need to access them to effectively advance science and bridge the gap between laboratory discovery and field applications. It has been shown that open data can empower research and drive agricultural innovation, which in turn helps address global societal and environmental challenges, and supports economic growth (GODAN 2016, 2018; Cowan et al. 2014). There is no denying that data become valuable when adhering to the FAIR principles^1^—findable, accessible, interoperable and reusable—with the intent to enhance the ability of machines to automatically find and use the data, in addition to supporting its reuse by individuals (Wilkinson et al. 2016). Yet, decisions and consensus with respect to practical implementation of these principles have the potential to limit adoption; the absence of proper standardization policies can block effective data exchange and integration (Krajewski et al. 2015; Jeppesen et al. 2018).

This article reports on a survey of experts that investigated potential uses and issues regarding the data relevant to the development and approval of new crop traits and agriculture in general. Our findings contribute to the debate about the fundamental principles for data sharing and related practical implementations. The article provides an overview of the different types of data, underlying legal protection mechanisms and issues around data sharing that are present in the context of the agriculture industry.

## Data types in the agri-food industry

The complex ecosystems in which agricultural production takes place needs to be better understood if agricultural productivity is to continue increasing. A better understanding of the dynamics can be obtained with modern monitoring instruments that continuously register changes in physical environmental parameters (Huang et al. 2018; Kamilaris et al. 2017). Agricultural ecosystems produce vast amounts of data that modern digital technologies can now register and measure. Mining, or finding patterns in, this vast amount of data can provide unique insights into how complex agricultural ecosystems function. In essence, agricultural production systems can be understood as, or reduced to, information flows.

As various types of data can be generated and captured from operating machines (sensor-equipped) or agricultural production processes themselves, related analytics are increasingly integrated in each stage of the agricultural value chain. Along with phenotypic and genomic data, modern plant breeding teams extensively use spatial data from mobile computing devices. Among the wide variety of data collected in modern plant breeding processes, we identify and define the following as the most significant:

- *Farm metadata* comprising management information (practices and technologies) such as seeding depth, cultivar, crop rotations, machinery diagnostics, time and motion,^2^ and the dates of tillage, planting, scouting, spraying, and input application.
- *Genomic data* related to the structure, function, evolution, mapping and editing of the genome (hereditary information in the form of DNA and RNA). Genotyping solutions have increased the efficiency of plant breeding in fields by enabling selection at the seedling stage before the trait of interest is expressed, yielding time, labor, space, and cost savings in bringing new varieties to the market (Kuriakose et al. 2020). Various environments and software packages with specialized features have been developed to digitize genomic data (Kuriakose et al. 2020).
- *Phenotypic data* related to the morphological and functional plant traits (growth, tolerance, resistance, architecture, physiology, yield, etc.) and the relationship between these functions plays a crucial role in selection decisions in plant breeding. Traditional phenotyping techniques along with available genetic information do not yield an in-depth functional analysis between genotype and phenotype, an obstacle to understanding the genetic basis for complex agronomic traits and thus, to progress in molecular breeding research (Rahaman et al. 2015; Omari et al. 2020; Zhao et al. 2019). With novel imaging technologies, reliable, automated and high through-put phenotyping or phenomics emerged to accelerate the accuracy and speed of phenotypic data for modelling and prediction of plant growth and structural development (Haque et al. 2018). Relative to genomics, digital phenotyping occurs at a slower pace as plant breeders are unsure on how voluminous and diverse phenotypic data can be usefully incorporated in breeding programs (Zhao et al. 2019; Awada et al. 2018; Kuriakose et al. 2020). Artificial intelligence tools are required to advance image-based phenotyping (Zhao et al. 2019). Current phenotypic data collection protocols remain largely fragmented and there is no standard way with which to store phenotypic both at the regional and the global levels, which is a challenge for data sharing (Zhao et al. 2019).
- *Logistics data* report on the transportation and storage of goods from the point of origin (farm) to the point of consumption (table). These data enable the traceability of product ingredients (Jin et al. 2017). The goal of logistics data is to meet end-users’ traceability and/or source of origin requirements in a timely and cost-effective manner (block chain distributed ledgers can capture and report progress through the supply system). Consumers are able to trace the origins and processing of products and make purchasing decisions based on this information.
- *Geospatial data* refer to the locational, attribute and temporal information about objects, events, or phenomena that have a location on the surface of the earth (Stock and Guesgen 2016). In agriculture, geospatial data are the site-specific data usually associated with precision agriculture (e.g. site-specific soil characteristics, inputs and yield) (Coble et al. 2016). Jeppesen et al. (2018) have implemented open geospatial infrastructure for data management and analytics and showed how it enables interoperability of precision agricultural data that can be shared in standardized formats, visualized online at a low cost for both developers and consumers of the data.
- *Telematics data* are collected from machines and can be measured and viewed remotely using sensors, positioning systems and telecommunication technologies. Telematics data refers to data on the field equipment and machinery operating certain tasks. As telematics involve wireless data transfer, efficient utilization is contingent on a reliable wireless internet connectivity (Mark and Griffin 2016).
- *Consumption data* refer to all information that pertains to consumption trends, such as tastes, packaging preferences, product labeling, appropriate presentation sizes, etc.

Massive high-dimensional data are being acquired from a gamut of sources throughout the multistage breeding process at low cost and in minimal time. Though reducing agricultural production systems and plants to their underlying information is a profound paradigm shift, this does not exempt this information from issues of access, intellectual property and privacy (Marden 2018; Smyth et al. 2020). The rise of digital networks expands data sharing and the risks of security breaches and misuse of sensitive or confidential data.

## Data sharing

### Legal protection mechanisms

The explosion of readily available data has yielded numerous benefits, including advancing research and speeding up innovation (by significantly reducing repetitive work), promoting scientific transparency and reproducibility, and stimulating new forms of collaborative knowledge production (e.g. citizen science, crowdsourcing) (Doldirina et al. 2018; Janssen et al. 2017). For instance, the Consultative Group on International Agricultural Research (CGIAR) is using crowed-sourced farmer knowledge to drive its Climate Change and Food Security (CGAFS) project (Bronson 2019). Public benefit, professional gain (scientific merit, partnership, etc.) and reproducible science were found to be the main motives for experts to share data. Despite these promises, the culture of data sharing remains fragile in domains where security and regulatory issues are prevalent. Privacy and transparency compete—sharing requires careful specification as unprotected disclosure can be risky. Data producers and owners use various measures to ensure lawful access to protect intellectual property assets. The most common of these legally-binding mechanisms are defined here.

- *Free accessibility* Governments around the globe are increasingly sharing publicly funded data on web portals and platforms free of charge, without restrictions on its usage or distribution, and in machine-readable formats, such as through the open government data movement (Zhenbin et al. 2020; OECD 2019). This is similar to open-source software development, where the code for software is publicly accessible and free to download. The only requirement with open-source development is that any specific improvements must be uploaded to the open-source sharing platform.
- *Contracts* traditional legal contracts (paper-based) define the rules and penalties in an agreement between two parties and, require central authority or external enforcement system (Brousseau and Glachant 2002, p. 3). Different parties (farmers, the cloud service providers, the networking service providers, etc.) are involved in contracts that include privacy, security and intellectual property protection clauses that need to be carefully negotiated to identify rights and obligations (Gupta et al. 2020). One recent innovation is computerized or smart contracts, which Szabo (1996) defines as “a set of promises, specified in digital form, including protocols within which the parties perform on these promises”. Smart contracts are being used to manage data and service sharing among users without third party involvement (Sultana et al. 2020). As self-executing tools, they could also be used to automate regulatory activities (e.g. reporting and monitoring of required data, checking for compliance and fining for noncompliance, and recording decisions by a regulator) (Magazzeni et al. 2017).
- *Intellectual property rights (IPRs)* are laws that establish a regime for access, use or reuse of data, metadata, or data products and include patents (exclusive rights in invention), trademarks (brand protection), copyrights (authorship/ownership protection), and trade secrets (proprietary or confidential information protection) (Doldirina et al. 2018). IPRs yield exclusive rights to the creators or inventors which encourages them to share information and data without fear of intellectual theft.
- *Encryption* refers to the use of cryptography techniques to transform a plain text database into a (partially) encrypted database, thus making it unreadable to anyone except those who possess the knowledge of the encryption key(s).^3^ This allows users to securely share data over an insecure network or storage site (Boneh et al. 2011, p. 253).
- *Commons* refers to institutions that manage access to shared resources under certain restrictions. Creative Commons (CC)^4^ is a US-based non-profit organization that offers a suite of licenses defining standard options for the distribution and re-use of creative, copyrighted works (Hagedorn et al. 2011). Being the most widely common-used licenses, CC are in use throughout the globe and supported by IPRs laws (Doldirina et al. 2018). CC licenses are commonly used to provide open access for the publication of journal articles.

While these protection mechanisms are globally adopted, requirements, scope and implementation vary among nations and jurisdictions, and dispute settlement (via litigation or other action) is complicated and far from certain (Doldirina et al. 2018). The management of data sharing remains limited in practice (Feasey and de Streel 2020). Scholars assert that unsynchronized principles—technical and legal—of data sharing and protection impede the interoperability of data and slow innovations in plant breeding.

### Governance of data sharing

As advanced earlier, data integration (e.g. linking genotypic and phenotypic information) presents one of the main—if not the greatest—challenges facing modern plant breeding community, both in academia and industry. Standardization of big data annotation and access is a solution (Kuriakose et al. 2020; Coppens et al. 2017). Taking the example of phenotyping, Coppens (Coppens et al. 2017, p. 62) posits that: “[t]he future of plant phenotyping lies in synergism, as the comprehensive integration and analysis of this ‘Big Data’ allow to unravel the biological processes governing plant growth and development, and to advance plant breeding for much-needed climate-resilient and high-yielding crops”. Similarly, Kuriakose et al. (2020) assert that the success of modern plant breeding depends on standardized data management to ensure harmonization of multidimensional data (like genomics, phenotypic, and environment).

In addition to technical data standardization (e.g. data description, formats, platforms), harmonization of processes and rules for data access and application of novel biotechnologies are also critical for crop improvement. The international scientific community is working through the CGIAR centres and DivSeek International Network (DIN) to develop some of these structures. Nationally, greater congruence between regulatory approaches to gene editing are expected to advance plant breeding research, enable trade, and offer novel products to consumers.

## Method

The data for this article stem from an online survey conducted between March and September of 2020. The survey was designed to gather expert opinions about the importance of different types of data in the agri-food industry and the legal mechanisms used to protect and manage use. The instrument also explored how countries might learn from each other when it comes to the approval of plant gene editing. The survey was emailed to a panel of 450 international scientists, government officials, and agribusiness professionals involved in plant biotechnology.^5^

This survey concludes a multi-year survey project piloted by a research team at the University of Saskatchewan between 2015 and 2020. The project investigated expert opinions regarding the application of new plant breeding techniques as a way to aid in the pursuit of global food security. Earlier surveys studied the regulatory and social barriers pertaining to novel breeding approaches using gene editing and related risks and potential benefits (Lassoued et al. 2018b, 2019a, b, 2020). As plant breeding has become data intensive, scientists are increasingly working on databases rather than cells. As part of our investigation, we explored (here and in a previous survey) how big data and related applications contribute to agricultural research productivity in ways that might enhance food security. This survey builds on a previous survey that explored how big data are currently used, benefits expected in the medium term, issues likely to arise in the data-sharing process, and impacts artificial intelligence could have on agriculture. This last survey of the project draws inferences on the enabling and disrupting impacts of technological innovations associated with big data.

Our study was deemed exempt from full ethics review by the Behavioural Ethics Board at the University of Saskatchewan on January 28, 2020 on the basis that the participants, as experts, were not themselves the focus of the research (BEH 97).^6^ Nevertheless, our survey presented participants with a standard consent statement describing the study, identifying the absence of known risks associated with participation, and a reminder that participation was voluntary and responses would be anonymous and confidential. Upon expression of consent, participants were presented with the questionnaire (provided in the Appendix).^7^

## Results and analysis

The survey was completed by 83 participants, resulting in a response rate of 18.5%. Respondents are predominantly males (75%), aged between 45 and 65. Forty-three percent of the participants reside in North America (NA), 31% in Europe, and 26% are from the rest of the world (5% in Africa, 5% in Asia, 9% in Oceania and 7% in Central and South Americas). Thirty-four percent work in industry or for a private research institution, 26% for an academic institution, and 28% for government or in a public research institution. Sixty-three percent identified themselves as scientific experts, and 24% as social experts (lawyers, agribusiness professionals, etc.).

### Data governance

Participants were asked to rank different types of data for their expected impact on enhancing food security. As illustrated in Table 1, genomic data (47%), farmer metadata (47%) and phenotypic data (44%) were highly rated by respondents. While tied with farmer metadata, genomic data received the highest number of first choice responses. Data Bridge Market Research forecasts the plant genomics market is projected to reach US$11.7 billion by 2025, a growth rate of 8.3% per annum.^8^

**Table 1:** Expert ranking of types of data by their importance to food security (% responses)

Types of data	Score (%)^a^
Genomics data	47
Farmer metadata	47
Phenotypic data	44
Logistics data (farm to table)	39
Geospatial data (soil and yield)	38
Consumption data	37
Telematics data (farming machinery diagnostics, time and motion)	28

^a^The score is a weighted sum value (%) of the 7 ranked responses where 1st, 2nd, 3rd, 4th, 5th, 6th and 7th choices were weighted 0.7, 0.6, 0.5, 0.4, 0.3, 0.2 and 0.1, respectively

Experts were asked which type(s) of data raise the most security or privacy concerns for them. Table 2 identifies that farmer metadata followed by consumption and genomics data are considered the most critical data types, identified by 37%, 27% and 25% of surveyed experts respectively. Big data governance including data ownership, privacy and security were identified as key requirements for reliable modern farm management (aka smart farming) (Gupta et al. 2020; Wolfert et al. 2017). Leakages of data produced from the mix of sensors, devices and farm equipment through unlawful access were judged to pose some threats. For example, Gupta et al. (2020 p. 34,569) identify that “leakage of agriculture anti-jamming devices information can help an attacker bypass these security measures, while leakage of soil, crop, and agriculture purchase information can cause severe economic losses to farmers, if such information is used by competitors or hostile actors”.

**Table 2:** Expert opinion regarding data security concerns (% responses)

Types of data	Score (%)^a^
Farmer metadata (geospatial soil/yield data linked to input used)	37
Consumption data	27
Genomics data	25
Logistics data (farm to table)	21
Telematics data (farming machinery diagnostics, time and motion)	19
Phenotypic data	16
Geospatial data (soil and yield)	12

^a^Total does not add up to 100% as the task is multiple response

### Open versus closed data management mechanisms

Most surveyed experts opined that consumption data (80%), followed by phenotyping data (69%), geospatial data (64%), genomics data (61%) and logistics data (58%) should be open and freely available to users (Table 3). As for farmer metadata and telematics data, experts had a diversity of opinions: some think that those data should be open, others think they should have restricted access, about one-quarter are uncertain.

**Table 3:** Expert opinion on data openness (% responses)

Data type	Should be open				Should be closed				Do not know				χ^2^_(df=4)_; p-value
	P	A	G	Total	P	A	G	Total	P	A	G	Total	
Consumption	33	20	27	80	4	4	4	12	4	4	0	8	4.088; 0.394
Phenotyping	25	20	24	69	15	4	5	24	1	4	2	7	6.444; 0.168
Geospatial	24	18	22	64	14	5	4	23	5	4	4	13	4.370; 0.358
Genomics	20	21	20	61	17	5	5	27	4	5	3	12	9.189; 0.057
Logistics	26	17	15	58	7	7	9	23	8	5	6	19	1.929; 0.749
Metadata	16	16	9	41	19	8	12	39	6	6	8	20	4.370; 0.358
Telematics	17	15	8	40	15	7	13	35	8	8	9	25	4.103; 0.392

P, A and G refer to the private, academic and government institutions

The cross tabulation—a joint frequency distribution of cases based on two or more categorical variables that can be analyzed with the Chi-square statistic (χ^2^_(df=k)_ with k degrees of freedom)—is used to determine whether the variables are statistically independent or are associated. Regardless of their workplace (private, academic or government), there was no statistically significant results (p-value > 0.05) in expert opinion regarding the openness of all types of data.

We also probed respondents about the impacts of open data. Open data are expected to generate a panoply of positive impacts on research transparency, food safety, pest management, and collaboration while there was concern about the impact on breeders’ revenue, in particular (Table 4). For the different impacts listed in Table 4, expert opinion on the impact of open data was not associated with the nature of their workplace (p-value > 0.05).

**Table 4:** Expert opinion on the impact of open data (% of responses)

	Negative	No impact	Positive	Don’t know
Transparency in research systems	1	13	81	5
Food safety concerns	3	10	79	8
Pest management	2	10	75	13
Collaboration^a^	10	9	70	11
Regulatory compliance	8	12	70	10
Innovation in any aspect of the plant breeding process	14	4	69	13
Claims about product quality	10	10	69	11
Management of scarce natural resources	6	16	65	13
Farmer profitability	12	18	53	17
Direct cash costs associated with accessing data	17	16	51	16
Indirect costs of data accessing data	15	21	51	13
Breeder’s revenue	35	19	26	20

^a^Collaboration between governments, businesses, non-governmental organizations (NGOs) and individuals

Our respondents reported they or their organizations use a mix of data management mechanisms, including contracts (51%), free accessibility (49%), copyrights (40%) and trade secrets (40%). Private research institutions are much more likely to use contracts (60%) compared with academic institutions (23%) and public research institutions (17%). The Creative Commons is used by a minority of respondents. While the majority of respondents thought most data should be open access (Table 3), only 38% of private researchers, 32% of academics and 30% of public research institutions offered free accessibility to others. In their place, they used copyrights (47%, 28% and 25% respectively) and trade secrets (59%, 25% and 15% respectively). Encryption is also used and respondents suggested they are considering using it more. Interestingly, at least one-quarter of the sample are not knowledgeable about the mechanisms used in their work environments, which generates potential risk of researchers not conforming to a practice out of ignorance (Table 5).

**Table 5:** Expert use of data management mechanisms in their work environment (% responses)

	Already using	Would consider using	Would not use	Don’t know
Contracts	51	17	6	26
Free accessibility	49	16	8	27
Copyrights	40	16	17	27
Trade secrets	40	19	12	29
Encryption	30	27	7	36
Creative commons licenses	23	27	8	42

### Data sharing

The vast majority of surveyed experts indicated that they, or their organization, would share their own created data with regulators (73%), international (69%) and national (62%) seed banks, public university breeders (69%), research organizations (67%) and online data repositories (58%) (Table 6). Access to and data sharing of research data have been encouraged by research institutions, journals and funding agencies, some of which adopted open access data policies (digital, online, free of charge, and free of most copyright and licensing restrictions) to encourage researchers to deposit underlying data in a disciplinary or institutional repository (National Academy of Sciences 2009). However, data access policies are still immature. Research institutions and sponsors may need to come together to identify best practices and policy models (National Academy of Sciences et al. 2009). The exception to this sharing is that few of the respondents judged it likely that they would share data with supply chain integrators (30%), farm implement manufacturers (30%) or multinational seed companies (27%).

**Table 6:** List of sources with whom experts share data (% responses)

	Unlikely	Neither	Likely	DNK
Regulators	4	5	73	18
International seed banks	6	9	69	16
Public breeders at universities	3	12	69	16
Consultative Group on International Agricultural Research Centres	7	10	67	16
Agriculture research organizations, agencies and departments	5	11	67	17
Environmental stewardship programs	8	11	65	16
National seed banks (e.g. USDA)	8	11	62	19
Online data repositories (e.g. genomics, phonemics websites)	12	13	58	17
Global Open Data for Agriculture and Nutrition	8	9	52	31
Small or entrepreneurial companies	17	23	40	20
Supply chain integrators (e.g. Cargill)	17	32	30	21
Implement firms (e.g. John Deere)	19	27	30	24
Multinational seed companies (e.g. Bayer)	29	28	27	16

One common perception is that increased data might complicate already difficult regulatory processes. To test that possibility, we asked our respondents whether they would be willing to share data created with regulators, finding that 73% of respondents were likely or very likely to do so regardless where they reside (p-value > 0.05). Indeed, the plurality of participants from North America (25%), Europe (25%), Oceania (7%), Africa (5%), Asia (5%) and Central and South America (5%) indicated that they have no issues to share data with regulators. Few if any of our respondents signaled much active concern with sharing, although about one third of respondents in North America hesitated to signal any clear intent.

### The role of foreign evidence in approval of gene editing

Survey respondents were presented with the following scenario: “In another country with a similar regulatory system to your own, a gene-edited crop or product received approval. This approval was based on a docket of evidence generated in that country (e.g. scientific experiments to support reviews for safety, efficacy and environmental sustainability)” and asked whether they believed their government would consider that same docket of evidence when applying for approval in their country. The responses (Table 7) show that 45% (including half of the North American and a third of the European participants) were optimistic that foreign data could satisfy domestic requirements, but decisions about approving a gene-edited crop would be made domestically. Almost one-quarter of the sample believed that data would be treated as supplemental and not sufficient for domestic requirements while 20% confirmed that data collection and analysis would need to be redone in their country. None of the experts expected that data and/or foreign decisions would satisfy domestic requirements. This result is not surprising given the heterogeneous regulatory systems and different environmental conditions across the world. Governments are unwilling to appear to be ceding regulatory authority to foreign governments or institutions. Over the last five years, a few countries in South America (esp. Argentina, Chile, Brazil and Colombia), North America (US and Canada), Australia and Japan have developed their own gene editing frameworks based on the risks of the resulting product rather than the process used to create it. The EU is a notable exception, as it treats gene editing as analogous to genetic engineering, and hence covered by de facto bans across the EU. Recently, many European research institutes and academies called for harmonization of the EU regulatory landscape, emphasizing that legislation on gene editing should consider the characteristics of the plant instead of the approaches used in its development (Dima et al. 2020).

**Table 7:** Experts opinion on how their government would use foreign evidence (% responses by region)

Would your government consider that same foreign docket of evidence when approving gene-edited crop in your country?	Africa	Asia	Europe	Central/South America	North America	Oceania	Total
No, data collection and analysis would need to be redone in my country	2	2	10	2	4	–	20
Yes, but data would be treated as supplemental and not sufficient for domestic requirements	2		6	1	11	4	24
Yes, foreign data could satisfy domestic requirements, but decisions would be made domestically	1	2	11	4	22	5	45
Don’t know	–	1	4	–	6	–	11
Total	5	5	31	7	43	9	100

When asked about how long it will take gene editing to have a significant impact on the agricultural sector, 88% assert that the impact will be seen in the global market (excluding the EU) within 10 years (Table 8). About half of the sample expect an impact will not be seen until later in the EU market.

**Table 8:** Expert opinion on the expected timeframe for gene editing to have a significant impact on the agricultural sector (% responses)

	< 5 years	5–10 years	10+ years	Don’t know
In your country	22	40	33	5
In the global market excluding the EU	30	58	11	1
In the EU market	6	22	49	23

## Conclusion

Experts think that genomic and phenotypic data along with farm metadata are most likely to impact future global food security. However, experts acknowledge that privacy issues, particularly around farm metadata, cannot be dismissed and must be appropriately addressed. Especially given that in the near future, it can be possible for ‘hackers’ to gain access to proprietary farm data. Such data can be sold or used with ill intent and end up being pernicious to farmers or entire industries. For several stakeholders in the agri-food sector, big data present several challenges including storage, management, integration, security and confidentiality. In addition, proprietary integrations and non-standardized formats and connections have been slowing the adoption of novel agricultural technologies (Jeppesen et al. 2018).

If data are going to drive the future of agriculture and food production, clear and transparent rules and customs for the access and mining of data are needed. A majority of surveyed experts, whether working in a private or a public institution, think open science and data sharing are beneficial overall, however technical and legal solutions such as collaborative infrastructure and coherent protection services have yet to be generalized. Moreover, though most experts think consumption data should be freely available, this is the second lowest ranked type of data that are believed to contribute to future food security. This is not surprising given that the respondents of this survey are predominantly scientists. That is, they are considering only supply side food security, and not contemplating demand. Hence, scientists are more willing to share information, which is not directly linked to their research endeavors, rather, the information that guides them. We highlight that the results reported here are based on the opinions of a relatively small expert sample. Further studies focusing on these issues are important.

## Data Availability

The raw data supporting the conclusions of this manuscript are not publicly available because academic survey policy at the University of Saskatchewan states that all survey data will be protected and held confidential to ensure responder anonymity. Requests to access these datasets should be directed to Dr. Stuart Smyth at stuart.smyth@usask.ca.

## Appendix: Questionnaire

Dear Participant,

- First, we would like to THANK YOU for your active participation in, and valuable inputs to, our multi-year survey project during which we have been collecting expert opinions on innovative technologies pertaining to plant breeding. Project output to date is available at https://research-groups.usask.ca/nbt-regulation/publications/articles.php. We have shared a number of papers with those who requested them. If you wish to receive full papers, please email us at nbt.regulation@usask.ca.
- Our project wraps up with the current invitation to participate in our eleventh and last survey. We would like to learn about your opinions pertaining to potential impact of digital agricultural knowledge on plant breeding regulations along with your overall views of our surveys.
- Dr. Stuart Smyth (stuart.smyth@usask.ca, (306) 966-2929) and Dr. Peter Phillips (peter.phillips@usask.ca, (306) 966-4021) have been leading this project over the course of the last four years. They can be contacted should you have any questions or comments. Any questions regarding your rights as a participant may be addressed to the University of Saskatchewan Research Ethics Office ethics.office@usask.ca; (306) 966-2975. Out of town participants may call toll free (888) 966-2975.
- This survey is hosted by Voxco, a Canadian-owned and managed company whose data is securely stored in Canada. Please consider printing this page for your records. There are no known risks to participating in this survey; however, as with any online activity the risk of breach of confidentiality is always possible.
- In order to complete this survey, you may be required to answer certain questions; however, you are never obligated to respond and you may withdraw from the survey at any time by closing your internet browser.
- By selecting next and completing this questionnaire, your free and informed consent is implied and indicates that you understand and accept the above conditions of participating in this study.

We are interested in your opinions about the potential uses and challenges around the use of data relevant to the development and approval of new crop traits and agriculture in general.

Q1. Which data do you think will have the biggest impact on enhancing food security?

Please rank the following type of data in order of importance with “1” being “data with the biggest impact” and “7” being “data with the least impact”.

- Geospatial (soil and yield) data
- Farmer metadata (Geospatial soil/yield data linked to input used)
- Telematics data (Farming machinery diagnostics, time and motion)
- Genomics data
- Phenotyping data
- Consumption data
- Logistics data (farm to table)

Q2. Which types of data raises most security/privacy concerns for you? (Check all applicable)

- Geospatial (soil and yield) data
- Farmer metadata (Geospatial soil/yield data linked to input used)
- Telematics data (Farming machinery diagnostics, time and motion)
- Genomics data
- Phenotyping data
- Consumption data
- Logistics data (farm to table)

Q3. For each type of data, please indicate whether you think it should be open (i.e., freely available to any user), or closed (i.e., restricted access):

**Table:**

Data types	Should be open	Should be closed	Don’t know
Geospatial (soil and yield) data			
Farmer metadata (Geospatial soil/yield data linked to input used)			
Telematics data (Farming machinery diagnostics, time and motion)			
Genomics data			
Phenotyping data			
Consumption data			
Logistics data (farm to table)			

Q4. For data you have created, which data management mechanisms do you or your organization use or consider using in future?

**Table:**

	Already using	Would consider using	Would not use	Don’t know
Copyright				
Trade secret				
Encryption				
Free accessibility				
Creative commons				
Contracts				
Other (please specify):				

Q5. Data is considered open if anyone is free to use, reuse, and redistribute it—subject only, at most, to the requirements of attribution and sharing.

In your opinion, what impact would open data have on the following considerations?

**Table:**

Impacts on:	Very negative	Negative	Neutral	Positive	Very positive	Don't know
Food safety concerns						
Management of scarce natural resources						
Pest management						
Transparency in research systems						
Innovation in any aspect of the plant breeding process						
Collaboration between governments, businesses, non-governmental organizations (NGOs) and individuals						
Direct cash costs associated with accessing data						
Indirect costs of data accessing data from others (i.e. time and resources it takes to locate, request and negotiate data access)						
Regulatory compliance						
Claims about product quality						
Farmer profitability						
Breeder’s revenue						

Q6. Would you or your organization share any data you created with any of the following:

**Table:**

	Very unlikely	Unlikely	Neither likely or unlikely	Likely	Very likely	Don’t know
Global Open Data for Agriculture and Nutrition (https://www.godan.info)						
Consultative Group on International Agricultural Research Centres (https://www.cgiar.org)						
National seed banks (e.g. USDA)						
International seed banks (e.g. The International Maize and Wheat Improvement Center https://www.cimmyt.org)						
National agriculture research organizations, agencies and departments in your countries or elsewhere						
Multinational seed companies (e.g.Bayer)						
Supply chain integrators (e.g. Cargill)						
Online data repositories (e.g. genomics, phonemics websites)						
Small or entrepreneurial companies						
Public breeders at universities (e.g. University of Wageningen)						
Environmental stewardship programs						
Regulators (e.g. European Food Safety Authority: http://www.efsa.europa.eu)						
Implement firms (e.g. John Deere)						
Other (please specify):						

Q7. Next, Please imagine the following scenario:

In another country with a similar regulatory system to your own, a genome-edited crop or product received approval. This approval was based on a docket of evidence generated in that country (e.g. scientific experiments to support reviews for safety, efficacy and environmental sustainability).

Do you believe that the government in your country would consider that same docket of evidence when applying for approval in your own country?

- No, data collection and analysis would need to be redone in my country
- Yes, but data would be treated as supplemental and not sufficient for domestic requirements.
- Yes, foreign data could satisfy domestic requirements, but decisions would be made domestically.
- Yes, data and foreign decisions would satisfy domestic requirements
- Don’t know

Q8. How many years do you think it will take for genome editing to have a significant impact on the agricultural sector?

**Table:**

	< 5 years	5–10 years	10+ years	I don't know
In your country				
In the global market excluding the EU				
In the EU market				

Finally, please tell us a bit about yourself …

Q9. Do you identify yourself as:

- Male
- Female
- Other
- Prefer not to say

Q10. Where do you currently reside?

- Africa
- Asia
- Europe
- Central or South America
- North America
- Oceania

Q11. Do you identify yourself as:

- A life scientist (biologist, ecologist, etc.)
- A social scientist/Agri-business professional (economist, lawyer, manager etc.)
- Other (Please specify):

Q12. Do you mainly work for:

- Industry/private research institution
- Academic institution
- Government/Public research institution
- Other (Please specify):

Q13. Any additional comments about this survey?

## Footnotes

1 https://www.go-fair.org/fair-principles/, https://www.force11.org/fairprinciples.

2 Refers to amount of time machinery has devoted to a task. If the machine moves, within-the-farm sensors indicate the paths taken.

3 https://link.springer.com/referenceworkentry/10.1007%2F978-1-4419-5906-5_677.

4 https://creativecommons.org/.

5 The surveyed expert panel was obtained from a contact database that was created using the emails of participants, of several conferences on biotechnology organized by the lead researchers over the past couples of decades, as well as of experts from online searches.

6 Per the Tri-Council Policy Statement: Ethical Conduct for Research Involving Humans, December 2014, Exemption Article 2.1.

7 Only questions for which results are reported in this analysis are shared in the appendix. As this survey concludes the multi-year survey project, questions that report on previous surveys and the whole project were not shared here as they are out of the scope of this article.

8 https://www.databridgemarketresearch.com/reports/global-plant-genomics-market; https://www.globenewswire.com/news-release/2019/09/18/1917201/0/en/Global-Plant-Genomics-Market-Analysis-by-Objective-Type-Trait-Application-and-Region-Forecast-2025.html.
